# Putting Like a Pro: The Role of Positive Contagion in Golf Performance and Perception

**DOI:** 10.1371/journal.pone.0026016

**Published:** 2011-10-20

**Authors:** Charles Lee, Sally A. Linkenauger, Jonathan Z. Bakdash, Jennifer A. Joy-Gaba, Dennis R. Profitt

**Affiliations:** 1 Department of Psychology, University of Virginia, Charlottesville, Virginia, United States of America; 2 Max Planck Institute for Biological Cybernetics, Tübingen, Germany; 3 Department of Psychology, University of Utah, Salt Lake City, Utah, United States of America; Royal Holloway, University of London, United Kingdom

## Abstract

Many amateur athletes believe that using a professional athlete's equipment can improve their performance. Such equipment can be said to be affected with *positive contagion*, which refers to the belief of transference of beneficial properties between animate persons/objects to previously neutral objects. In this experiment, positive contagion was induced by telling participants in one group that a putter previously belonged to a professional golfer. The effect of positive contagion was examined for perception and performance in a golf putting task. Individuals who believed they were using the professional golfer's putter perceived the size of the golf hole to be larger than golfers without such a belief and also had better performance, sinking more putts. These results provide empirical support for anecdotes, which allege that using objects with positive contagion can improve performance, and further suggest perception can be modulated by positive contagion.

## Introduction

In a host of activities, people think that they will perform better when their equipment has been previously used by an admired professional. For example, in the 2002 film, *Like Mike*, a young boy discovers a pair of Michael Jordan's basketball shoes, which impart extraordinary basketball talent to the wearer [1]. But anecdotes of this phenomenon are not limited to fiction. Philosopher Eugen Herrigel recounted his experience of the notoriously difficult art of Zen archery:

If I had been continually shooting badly, the Master gave a few shots with my bow. [My] improvement was startling: it was as if the bow let itself be drawn differently, more willingly, more understandingly. This did not happen only with me [2].”

Indeed, many sports enthusiasts believe that using a professional's equipment can confer upon them performance benefits. For example, one might believe that they would have a higher batting average by using one of Mickey Mantle's baseball bats. Likewise, one might think that using golf legend Arnold Palmer's putter might lead to a lower putting average in a round of golf. Such seemingly superstitious beliefs are pervasive and consistent with the notion of *positive contagion*.

The rule of contagion states that “there can be a permanent transfer of properties from one object (usually animate) to another by brief contact [3].” Thus, contagion describes how contact with the object transfers its positive or negative properties to another object. For example, in a study on contagion effects, Rozin et al. [3] offered participants the choice between two glasses of juice and asked them to rate which juice they preferred. Afterwards, the experimenters took a sterilized dead cockroach and submerged it into one of the juice glasses. After pouring fresh glasses of the two juices, without the roach present, participants were asked to rate their desirability toward each juice. Participants rated the juice which previously contained the roach as less desirable, suggesting that the juice had been effectively “contaminated” in the minds of participants. Within the same study, Rozin et al. [3] also found that individuals rated the value of laundered blouses worn by liked individuals higher than those of disliked individuals. Together, these results suggest two things: that contagion can shape beliefs and that even a brief history of real *or* perceived contact is sufficient to elicit contagion effects. Nevertheless, it is unclear how contagion can affect the quality of the individual's *interaction* with the contaminated object. In this study, we examined whether golfers' positive beliefs about their sports equipment could affect their putting performance. Specifically, we investigated whether knowledge that a putter previously used by a famous golfer could lead to an improvement in a putting task involving that putter.

Perceptual processes could also facilitate a performance improvement. Several studies have demonstrated that task performance can influence the perceived size of the target relevant to the task. For example, those who were better at throwing tennis balls or darts to a target perceived the target as larger after throwing than those that were less successful [4], [5]. Similarly, improving performance by decreasing task difficultly has been shown to lead to an increase in perceived target size [6]. Likewise, better putting performance in golf was associated with perceiving the golf to be larger after the task [6].

In prior research, however, perception was always assessed *after* performance. Ordered as such, it is unclear whether (a) an increase in the perceived size of the target leads to better performance, (b) better performance leads to an increase in the perceived size of the golf hole, or (c) whether there is a reciprocal relationship between perception and performance. Thus, we examined whether positive beliefs about a putter could also affect the perceived size of the golf hole *prior* to putting. If the golf hole's perceived size is affected prior to performance, then it suggests that changes in apparent target size are not necessarily contingent on task performance and that such changes could beneficially influence performance. It also leaves open the possibility that factors unrelated to task performance (e.g., beliefs) can alter one's perception of target size.

As a result, we examined whether positive beliefs about a putter could affect the perceived size of the golf hole prior to putting and putting performance. We found that participants who used a putter they believed was previously used by professional golfer Ben Curtis perceived the golf hole as larger prior to putting and also performed better.

## Methods

### Participants

Forty-one right-handed undergraduates (93% men, *M* = 19.00 years old) at the University of Virginia who indicated having golf experience and following the PGA Tour participated for course credit. The study followed all institutional guidelines related to the protection of human participants; written informed consent was obtained from all participants. The institutional review board at the University of Virginia approved this study.

### Stimuli & Apparatus

**Figure 1:**
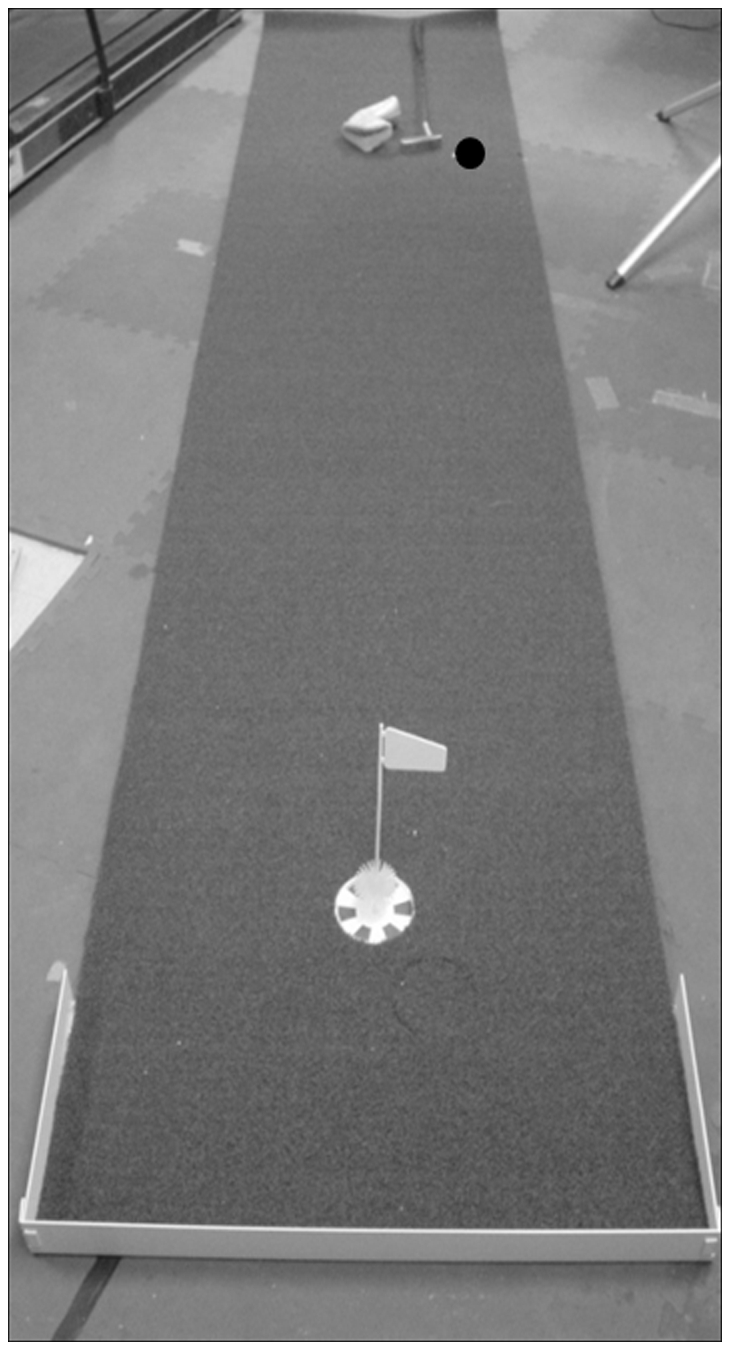
The putting mat used during the experiment. The black dot signifies the initial location of the golf ball.

Participants used an 89 cm Titleist Scotty Cameron Newport 2 putter matching the specifications of Ben Curtis' putter A golf hole (10.8 cm diameter) was in the center of the width of an artificial green putting mat (3.66×.76 m) with a Stimp rating of 10.5, see Figure 1. A *Stimp* rating is a measure of green speed; a higher rating corresponds to a faster speed. The 10.5 Stimp rating is considered fast and is generally the speed of greens professional golfers play on. An HP laptop (35.6 cm diagonal display) with the laptop keyboard and external mouse were used for golf hole size estimations. Hole size estimates were made using the elliptical drawing tool in Microsoft (MS) Paint.

### Procedure

Participants were randomly assigned to either the professional or control group. Prior to putting, participants completed a survey designed to assess experience and pre-manipulation confidence levels across groups. In it, participants reported their golf experience by indicating the number of rounds of golf (either 9 or 18 holes) they played in the past three months by circling the appropriate number range (i.e., *0–3, 4–7, etc.*). Then, they listed their level of confidence with their putting on a 6-point Likert scale (1 = not at all, 6 = very strongly).

After completing the surveys, participants in the professional condition were told that the researchers had acquired a putter formerly used by the well-known PGA Tour player Ben Curtis. Afterwards, they were asked extemporaneous questions (e.g., “Have you heard of Ben Curtis?”, “Isn't that cool?”) and also told about Ben Curtis' recent successes on the PGA Tour in order to convince participants and emphasize Ben Curtis' superb golf talent. This interchange between the experimenter and participant amounted to approximately 30 seconds in the 15 minute experiment. By contrast, control participants were not told anything about the putter's history.

First, participants viewed the golf hole from a distance of 2.13 m. With the laptop, participants used a mouse to control MS Paint's elliptical tool, estimating the size of the golf hole by drawing a circle on the computer screen which corresponded to the physical size of the golf hole. To promote accuracy, participants were encouraged to redraw the circle until they believed it matched the size of the golf hole. Then, to gain a feel for the speed of the indoor putting mat, familiarization with the weight, and correct individual grip height of the putter, participants attempted three practice putts from a distance of 2.13 m. Next, participants took 10 test putts. To increase difficulty, participants were asked to putt from an area that was not parallel to the major axis of the putting mat (see Figure 1). Otherwise, participants may have reduced the task difficulty by using the closest edge of the putting matt to facilitate alignment.

## Results

**Figure 2:**
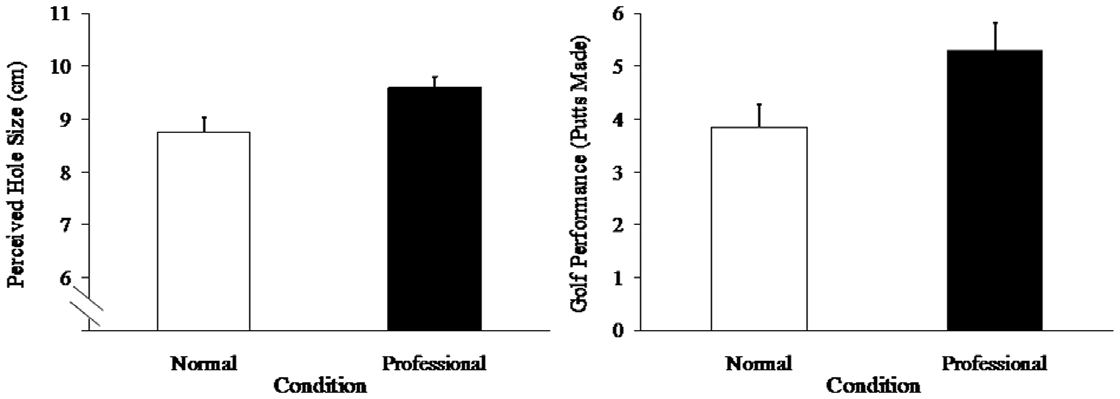
Perceived hole size before putting(a) and (b) putts made. Errors bars indicate one standard error of the mean.

The professional group perceived the golf hole to be bigger and sank more putts than the control group. Independent sample *t*-tests indicated the professional group perceived the golf hole to be larger (*M* = 9.60 cm, *SD* = .88) than the control group, (*M* = 8.75 cm, *SD* = 1.26), *t*(38) = 2.49, *p* = .02 (two-tailed), *d* = .79, see Figure 2a. In addition, more putts were made by the professional group (*M* = 5.30, *SD* = 2.36) than the control group (*M* = 3.85, *SD* = 1.95), *t*(38) = 2.11, *p* = .04 (two-tailed), *d* = .67, see Figure 2b. In assessing putting performance, *putt dispersion* was also recorded (see Text S1). This is a more precise measure of putting performance than the dichotomous make-or-miss method, see Text S1 for details. Putt dispersion analysis preserved the same relationship, *p*<.05 (two-tailed), *d* = .74. There was no difference between the professional and control group in golf experience or pre-manipulation confidence, *ps*>.47 (two-tailed). One participant was excluded on the basis of having no prior golf experience. Because golf experience typically leads to a consistent putting technique, it is difficult to assess how one without experience might benefit from positive contagion.

## Discussion

As hypothesized, the belief that one was using a professional golfer's putter led to perceiving the golf hole as larger and improved putting performance. Together, our results suggest that positive contagion can increase perception of golf hole size and improve putting performance.

These findings are consistent with Rozin et al. [3] suggesting that once an object (e.g., a putter) comes into contact with a positively appraised object (e.g., a professional golfer), a transference of positive attributes occurs. Our results extend Rozin's theory of contagion by demonstrating transference to perception and action associated with task performance. Moreover, instead of finding a post-performance perceptual change, as in previous studies [6], [7], the present findings illustrate a change in the apparent size of the golf hole that occurred *prior* to putting. This suggests that feedback on one's performance of the immediate task is not always a necessary condition for influences on perception.

Similarly, these findings allow for the possibility that increases in perceived target size can improve performance, because perceived hole size was influenced prior to task performance. However, it is also possible that a third variable could independently influence both perception and performance. Hence, our results allow for the possibility that the relationship between perception and performance is more complex than previously assumed. Indeed, actual task performance is not the only non-optic variable that can affect the perceived size of the task-relevant target.

There are several possibilities that could explain how positive contagion influenced putting performance. Previous research has shown that engaging in positive imagery before a sports competition is positively correlated with performance [8], [9]. In golf specifically, *pre-competition general mastery imagery* among collegiate golfers has been found to be positively correlated with performance; such imagery involves imagining oneself as having control over one's situation and engaging in a state of focus and mental toughness [9]. In the current study, participants were given a putter believed to have been used by Ben Curtis. It is possible that this may have encouraged the use of positive imagery such that they imagined Curtis' past successes, or at least, the sorts of positive affect associated that professional golfer's triumphs may induce in fans of golf.

Priming could also provide another theoretical basis for changes in performance. Priming involves a mental activation of certain stereotypes, which elicit corresponding behavior. For example, priming students with the term “professor” activates the concept of intelligence, thereby enhancing performance on subsequent knowledge tests [10]. Hence, believing that a professional used one's putter could have implicitly activated the concept of “skill” thereby improving putting performance.

Positive contagion might even be conceptualized as a placebo effect, a therapeutic effect resulting from belief and expectation [11]. Although inducing placebo effects typically requires using drugs or sham surgeries, beliefs alone can cause strong changes in health and physiological measures [12]. The belief that an individual is using “Ben Curtis' putter” could, in turn, enhance one's perceived putting capabilities. (Note that, in the present study, the manipulation was subtle and only concerned ownership of the putter.)

Here, one might object that using “Ben Curtis' putter” should not alter expected putting capabilities. To the contrary, prior work has shown that positive contagion can lead one to impute more value to an object [3]. Consequently, participants in the professional group may have assigned greater value to the putter and therefore amended their perceived putting abilities. This is consistent with the observation that object valuation may be rooted in irrationality and that ownership and an object's origin matter [13], [14]. Finally, object valuations can have powerful placebo effects. Ariely [15] demonstrated that the price of medication impacts its efficacy and, more relevantly, Damisch [16] observed that objects believed to be “lucky” facilitate better task performance. In short, object valuation and placebo effects, in tandem, help explain our findings.

This study demonstrates that positive contagion can improve putting performance. The belief that one is using a professional golfer's putter can elicited changes in performance and perception. Our findings also demonstrate that perceptual changes can precede task performance implying that, initially, other non-optic variables aside from actual task performance can influence the perceived size of the target. Lastly, we have proposed potential causal determinants of how contagion influences putting performance. And even while the role of imagery, self-efficacy, priming, and placebo effects is imprecise, this research lays significant groundwork for future studies exploring phenomena formerly considered superstitious. We would like to thank Brian Nosek for comments on earlier versions of this manuscript.

## Supporting Information

Text S1 **A justification as well as a description of how putt dispersion was measured.**
(DOCX)
