# Supplementary material for: Putting Like a Pro: The Role of Positive Contagion in Golf Performance and Perception
Source: PLoS One. 2011 Oct 20;6(10):e26016. doi: 10.1371/journal.pone.0026016 (PMC3197590; doi:10.1371/journal.pone.0026016)
Supplement: Text S1 — A justification as well as a description of how putt dispersion was measured. (DOCX) [file pone.0026016.s001.docx]

Supplemental Materials

Generally speaking, one might consider a golfer who consistently putts a ball within inches of the hole to have respectable putting skills. Thus, if such a golfer missed all putts, but came within inches of the hole, recording only the number of putts made might belie the golfer’s actual putting skills. Putt dispersion was measured by using a yard stick to measure the distance between the closest part of the ball to the nearest edge of the golf hole. Then, based on the distance, putts were placed into one of five categories ranging from “Hole,” which signifies a holed putt, to distances ranging from 0 to 30.5 cm in increments of 10.2 cm. If the part of the ball nearest the edge was exactly 10.2 cm, or any integer multiple thereof, from the closest edge of the hole, the putt was categorized according to whichever distance category contained the largest portion of the ball. The closer the category the more points the putt was awarded. Putt dispersion analysis also preserved this same relationship. Hence, participants in the professional group had higher putt dispersion scores (*M* = 7.76, *SD* = 1.43) than those in the control group (*M* = 6.80, *SD* = 1.16), *t*(38) = 2.34, *p* < .05 (two-tailed), *d* = .74.
